# Pneumococcal vaccination and primary care presentations for acute respiratory tract infection and antibiotic prescribing in older adults

**DOI:** 10.1371/journal.pone.0299924

**Published:** 2024-04-18

**Authors:** Fariha Binte Hossain, Sanjay Jayasinghe, Katrina Blazek, Wen-Qiang He, Bette Liu

**Affiliations:** 1 School of Population Health, UNSW, Sydney, Australia; 2 National Centre for Immunisation Research and Surveillance (NCIRS), Kids Research, Sydney Children’s Hospitals Network, New South Wales, Sydney, Australia; 3 Children’s Hospital at Westmead Clinical School, The University of Sydney, Sydney, Australia; Stanford University School of Medicine, UNITED STATES

## Abstract

**Background:**

While the 23-valent pneumococcal polysaccharide vaccine (PPV23) has demonstrated its role in preventing severe pneumococcal disease, its impact on more non-specific conditions like acute respiratory tract infection (ARI) and lower respiratory tract infections (LRTI) remains unclear. We aimed to investigate the role of PPV23 in prevention of presentations for ARI and LRTI and related antibiotic prescriptions among older adults in primary care.

**Methods:**

Using a nationwide general practice dataset, we followed a cohort of regularly attending patients aged ≥65 years from 1 January 2014 until 31 December 2018 for presentations for ARI, LRTI, and related antibiotic prescriptions. Associations between PPV23 receipt and each outcome were assessed using a multiple failures survival model to estimate hazard ratios (HR) adjusted for age, sex, socioeconomic status, and various health measures.

**Results:**

A cohort of 75,264 patients aged ≥65 years (mean 75.4, 56% female) in 2014 was followed. The incidence of presentations for ARI, ARI-related antibiotic prescription, LRTI, and LRTI-related antibiotic prescription was 157.6, 76.0, 49.6, and 24.3 per 1000 person-years, respectively. Recent PPV23 vaccine receipt was associated with a small reduction in ARI presentations (adjusted HR vaccinated vs. unvaccinated 0.96; 95%CI 0.94–0.98; p = 0.002); however, there was no reduction in ARI-related antibiotic prescription, LRTI presentation, nor LRTI-related antibiotic prescription (adjusted HR were 0.99[95%CI 0.96–1.03], 1.04[95%CI 0.99–1.09], 1.07[95%CI 1.00–1.14]).

**Conclusion:**

PPV23 vaccination in older adults may result in a small reduction in the incidence of total ARI presentations in primary care. However, the effect is small and residual confounding cannot be excluded.

## Introduction

Pneumococcal disease, caused by *Streptococcus pneumoniae*, can present as a heterogenous medical condition that ranges from mild respiratory tract infections such as bronchitis, rhinitis, acute sinusitis, otitis media, conjunctivitis to severe life-threatening invasive pneumococcal diseases such as meningitis and septicaemia [1]. The burden of pneumococcal disease varies substantially across geographical regions and by different age groups, with higher burden found particularly in young children and the elderly. Data from Australia’s National Notifiable Disease Surveillance System (NNDSS) indicates that in 2016 the crude notification rates for invasive pneumococcal disease were 188.9 per million among infants and 166.9 per million among individuals aged 65 years and above [2]. Systematic reviews suggest that the pneumococcal vaccines, 23-valent pneumococcal polysaccharide vaccine (PPV23), and 13-valent pneumococcal conjugate vaccine (PCV13) can protect against invasive pneumococcal disease and pneumococcal pneumonia in the elderly [3–6]. However, it is uncertain how much pneumococcal vaccines may prevent more nonspecific outcomes such as lower respiratory tract infections (LRTI) [7–9]. A large proportion of acute respiratory tract infections (ARI) lead to antibiotic prescriptions, with ARIs accounting for an estimated 50% of all antibiotic prescriptions in adults [10–12]. As a reduction in antibiotic use is likely to help to curb the global emergence of antimicrobial resistance, it is also important to quantify whether pneumococcal vaccination has any role in reduction of ARI presentations and subsequently ARI-related antibiotic prescriptions, and no previous study has yet investigated this in adults.

In Australia, similar to many high-income countries, pneumococcal vaccines are routinely recommended for specific groups such as young children, older adults, and individuals with certain at-risk conditions to prevent pneumococcal disease [13]. Since the introduction of the universal infant PCV vaccination program in 2005, vaccine coverage in eligible children has been consistently over 90% [14]. A systematic review analysing Australian studies from 1992 to 2013 estimated that pneumococcal vaccination coverage among individuals aged 65 and above ranged from 50.3% to 72.8% [15]. Australia’s pneumococcal vaccination program has evolved significantly over time [15], with the most recent changes implemented on 1 July 2020 [13]. Prior to this, in 1997 adults aged 65 years and above were recommended to receive at least one dose of PPV23 with funding of the single dose available since 2005 [16]. This study aims to estimate the association of PPV23 vaccination with ARI and LRTI presentations and ARI- and LRTI-related antibiotic prescription in primary care settings among older Australian adults.

## Materials and methods

### Data source and study population

MedicineInsight is a de-identified longitudinal research database derived from national electronic health records for over 3.6 million patients in approximately 600 general practices (GPs) across Australia. A detailed description of MedicineInsight has been published elsewhere [17]. Australians have universal health coverage including access to GPs and patients are free to visit multiple GPs of their choice. The majority of vaccines included in the National Immunisation Programme (NIP) for children and adults aged 65 and above are administered through general practice [18]. We used a 25% random sample of patients in the database (the maximum allowable for external researchers), which represented 573 general practice sites. Information extracted included patients’ demographics, reasons for encounters (free-text), diagnoses (free-text), prescription items, vaccination history, and practices’ geographic information. We only included data from GPs that met practice quality criteria each year from 2012 to 2018 as described previously [19]; this was GPs with annually (a) ≥250 encounter records; (b) ≥300 prescription records; (c) ≥100 unique patients who visited the practice; and (d) <15% of prescription records with missing medicine names. Ethical approval for the study was granted by the University of New South Wales Human Research Ethics Committee (approval no. HC200176). The data were accessed on 23 June 2021 for research purposes.

**Fig 1:**
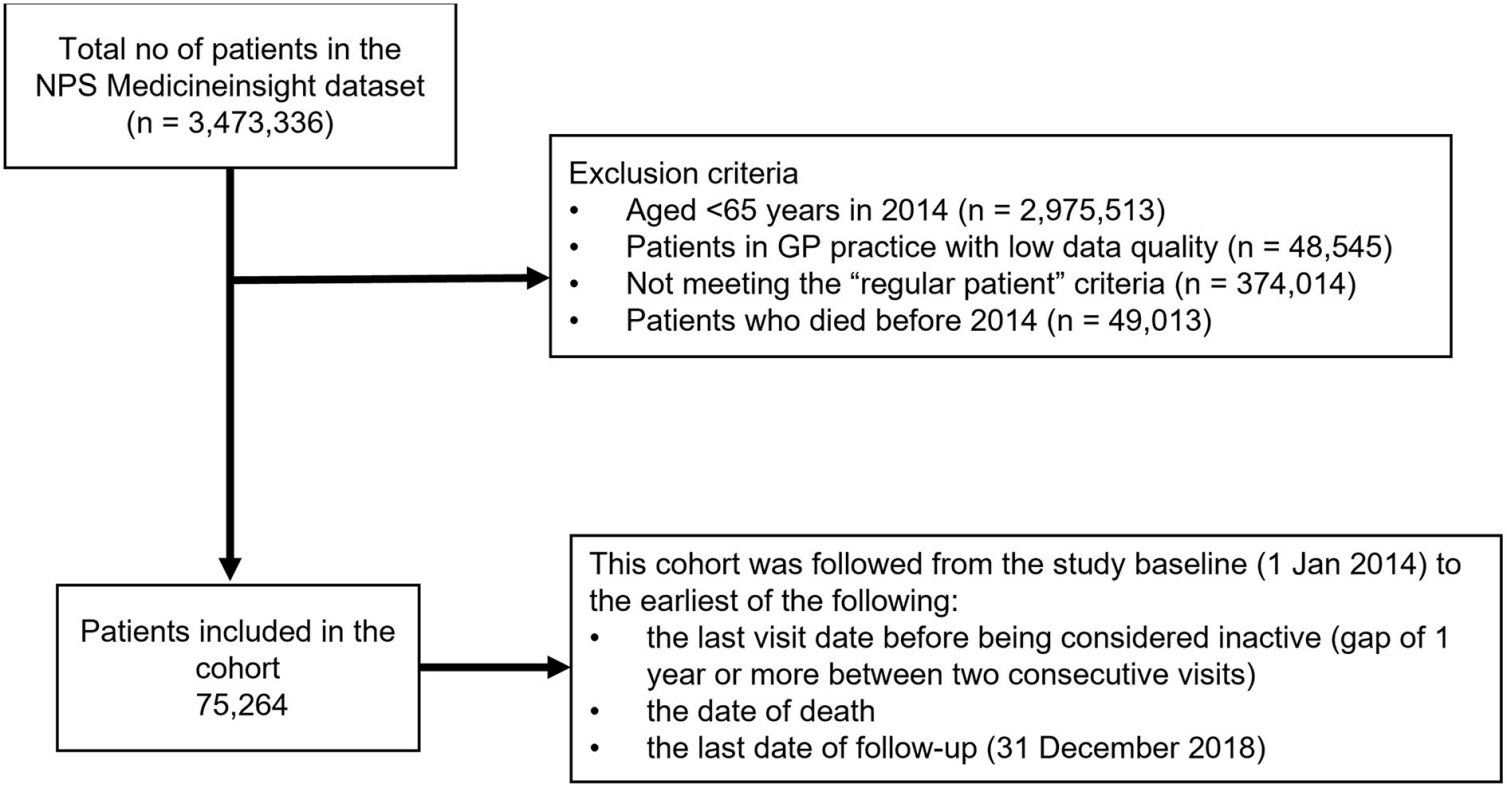
Flow chart of study cohort selection.

A retrospective cohort study design was used to assess the associations of PPV23 vaccination with ARI, ARI-related antibiotic prescription, LRTI, and LRTI-related antibiotic prescription. We defined a “regular attender” as a patient who attended the same GP at least once in each of the preceding two years (e.g., 2012 and 2013) [19]. We included all patients aged ≥65 years in 2014 who were regular attenders. In Australia, individuals have the flexibility to choose and switch general practitioners at any time, therefore we limited our study to those who consistently attended. Patients recorded as inactive, deceased (before 2014), or with missing data on year of birth and sex were excluded from analyses (see Fig 1 for details). This 2014 cohort was followed through to 2018.

### Pneumococcal vaccination

Information on pneumococcal vaccination and date of vaccination was retrieved from MedicineInsight’s Immunisation dataset. We used vaccine brand names as search terms (Pneumovax or Prevenar 13) as well as a combination of all plausible terms (pneumo’ and ‘vax’, ‘vac’ or ‘injection’) to capture GP encounters related to pneumococcal vaccination (see S2 Table for details). In our cohort, 99.5% of those who received a pneumococcal vaccine received PPV23, 0.1% received PCV13, and 0.4% received an unspecified pneumococcal vaccine. Therefore, we focussed on quantifying the association of PPV23 with the outcomes and excluded the small number who received PCV13 and unspecified pneumococcal vaccine from analyses.

### Outcomes

Four outcomes were assessed—i) acute respiratory tract infection (ARI) events, ii) ARI-related antibiotic prescriptions, iii) lower respiratory tract infection (LRTI), and iv) LRTI-related antibiotic prescriptions.

For ARI and LRTI, we searched the “encounter reason,” “diagnosis reason,” and “prescription reason” data fields in the corresponding datasets from the MedicineInsight database using a combination of relevant medical terminologies and shorthand abbreviations. Search terms were based on and earlier study (19) and are provided in S3 Table. If a patient had multiple records of ARI or LRTI events within a 30-day period, these were considered part of the same event. Conversely, ARI or LRTI events occurring more than 30 days apart in the same patient were treated as recurrent events.

We identified antibiotic prescribing from the “medicine active ingredient” field of the prescription dataset using combinations of search terms for systemic antibiotics [19] commonly prescribed to treat respiratory illnesses (S4 Table). If patients had a record of an antibiotic prescription and a record of ARI or LRTI diagnosis on the same date, then they were considered to have had an ARI- or LRTI-related antibiotic prescription.

### Statistical analysis

To describe the cohort characteristics, we used proportions for categorical variables and means and standard deviation (SD) for continuous variables. For the longitudinal analyses, we followed patients from the study baseline (1 Jan 2014) to the earliest of the following: the last visit date before being considered as inactive, the date of death, or the last date of follow-up (31 December 2018). A patient was considered inactive if they had a gap of one year or more between two consecutive visits. We examined the distribution of each outcome as recurrent events and estimated an overall incidence rate with a 95% confidence interval (CI). The incidence rate was calculated by dividing the number of cases over the number of person-years accumulated during the follow-up period.

We treated pneumococcal (PPV23) vaccination status as a time-varying variable. Those with a record of PPV23 receipt between 2000 and 2013 (i.e., before baseline) were defined as vaccinated at baseline. Those with a first record of pneumococcal vaccination after the baseline (i.e., between 2014 and 2018) were initially classified as unvaccinated and then contributed person-time to the vaccinated category from 14 days after their date of vaccine receipt. We used the Prentice, Williams, and Peterson Total Time (PWPTT) multiple failures survival model to estimate hazard ratios (HR) with 95% CI for each of the four outcomes (ARI, ARI-related antibiotic prescriptions, LRTI, and LRTI-related antibiotic prescriptions) comparing vaccinated vs. unvaccinated patients. More details on how we dealt the data for time-varying vaccination status and multiple outcome events are given in S1 Methods in S1 File S1 Fig. Models were adjusted for potential confounders: age at baseline (categorized as 65–69; 70–74; 75–79; 80–84; ≥85 years), sex, socio-economic status of GP in quintiles (Index of Relative Socio-economic Advantage and Disadvantage (IRSAD) [20], a macroeconomic indicator of relative economic and social advantage/disadvantage position within an area/postcode compared to the rest of the country), the total number of GP visits in 2012 and 2013 (categorized as 2–10; 11–20; ≥ 21 visits), remoteness of practice (categorized as major cities; inner regional; outer regional/remote/very remote, based on the Accessibility and Remoteness Index of Australia [21], smoking status (categorized as a smoker; ex-smoker; never smoker), influenza vaccination status during the follow-up period (yes, no), and comorbidity status (i.e. ever recorded asthma, chronic obstructive pulmonary disease (COPD), diabetes, chronic heart disease, chronic kidney disease, chronic liver disease, and haematological malignancy). We used pre-coded conditions information available in the MedicineInsight data to define these comorbidities, except for haematological malignancies which was defined by free-text searching (more details in the S1 Methods in S1 File S1 Table).

We also assessed the association of PPV23 with the outcome of interest by time since vaccination (unvaccinated, vaccinated within <5 years of baseline, and vaccinated ≥5 years prior to baseline). We also stratified analyses by the presence of the specified comorbidities (yes, no) and by smoking status (ever smoker, never smoker). To examine the validity of our primary analysis, we undertook analyses with negative control outcomes, including gastroenteritis and urinary tract infection (UTI), using definitions from earlier studies [22, 23]. We also conducted a sensitivity analysis where we restricted follow-up to one year (from Jan 2014- Dec 2014) to reduce any potential confounding due to changes in age over time.

All analyses were performed using Stata V.17.1 (StataCorp, College Station, Texas, USA). All statistical analyses were two-sided, and a p-value <0.05 was considered statistically significant.

## Results

A total of 75,264 regular attendees aged a mean of 75.4 years at baseline were included. Over half (56%) of patients were female, and 58% came from practices in major cities. The majority of patients were never smokers (53.3%), and 27% had at least one of the listed comorbidities. Table 1. shows the baseline characteristics according to the record of receipt of at least one dose of PPV23 between 2000 and 2018. A total of 52,277 patients (70%) had received PPV23 vaccine in this time period. Among those who received at least one dose of PPV23, 60.8% had one dose, and 39.2% had two or more doses. Compared to those who did not receive PPV23, those who received the vaccine were more likely to be older, ex-smokers, and have at least one comorbidity. The vaccinated group also had more GP visits in the preceding two years and were more likely to have received an influenza vaccination during follow-up period.

**Table 1:** Characteristics of included patients aged 65 years or above at study baseline (2014) according to pneumococcal vaccination (PPV23) received during 2000 to 2018, Australia.

	Total	Never received PPV23	Ever received at least one dose of PPV23	p-value
	N = 75264	N = 22,987	N = 52,277	
Age at baseline (2014)				<0.001
65–69 years	21,599 (28.7%)	8,706 (37.9%)	12,893 (24.7%)	
70–74 years	17,374 (23.1%)	4,873 (21.2%)	12,501 (23.9%)	
75–79 years	14,382 (19.1%)	3,552 (15.5%)	10,830 (20.7%)	
80–84 years	10,711 (14.2%)	2,672 (11.6%)	8,039 (15.4%)	
85+ years	11,198 (14.9%)	3,184 (13.9%)	8,014 (15.3%)	
Sex				0.084
Male	33,222 (44.1%)	10,255 (44.6%)	22,967 (43.9%)	
Female	42,042 (55.9%)	12,732 (55.4%)	29,310 (56.1%)	
Socioeconomic status of GP (quintiles)				<0.001
Most disadvantaged	14,000 (18.6%)	4,331 (18.8%)	9,669 (18.5%)	
Disadvantaged	13,505 (17.9%)	4,464 (19.4%)	9,041 (17.3%)	
Middle	18,893 (25.1%)	5,429 (23.6%)	13,464 (25.8%)	
Advantaged	11,723 (15.6)	3,650 (15.9%)	8,073 (15.4%)	
Most advantaged	16,867 (22.4%)	5,004 (21.8%)	11,863 (22.7%)	
Missing	276 (0.4%)	109 (0.5%)	167 (0.3%)	
Remoteness of GP				<0.001
Major cities	43,523 (57.8%)	13,657 (59.4%)	29,866 (57.1%)	
Inner regional	22,525 (29.9%)	6,192 (26.9%)	16,333 (31.2%)	
Outer regional / remote / very remote	9,216 (12.2%)	3,138 (13.7%)	6,078 (11.6%)	
Smoking status				<0.001
Smoker	3,690 (4.9%)	1,357 (5.9%)	2,333 (4.5%)	
Ex-smoker	27,282 (36.2%)	7,234 (31.5%)	20,048 (38.3%)	
Never smoker	40,078 (53.2%)	11,965 (52.1%)	28,113 (53.8%)	
Missing	4,214 (5.6%)	2,431 (10.6%)	1,783 (3.4%)	
No. of GP visits in 2012–13				<0.001
2–10 visits	30,717 (40.8%)	11,825 (51.4%)	18,892 (36.1%)	
11–20 visits	23,922 (31.8)	6,458 (28.1%)	17,464 (33.4%)	
21 or more visits	20,625 (27.4%)	4,704 (20.5%)	15,921 (30.5%)	
Asthma	4,328 (5.8%)	1,057 (4.6%)	3,271 (6.3%)	<0.001
COPD	4,252 (5.6%)	890 (3.9%)	3,362 (6.4%)	<0.001
Heart disease	7,493 (10.0%)	2,043 (8.9%)	5,450 (10.4%)	<0.001
Diabetes	6,813 (9.1%)	1,768 (7.7%)	5,045 (9.7%)	<0.001
Chronic liver disease	124 (0.2%)	47 (0.2%)	77 (0.1%)	0.075
Chronic kidney disease	1,698 (2.3%)	379 (1.6%)	1,319 (2.5%)	<0.001
Haematological malignancy	245 (0.3%)	60 (0.3%)	185 (0.4%)	0.039
Any of the above comorbidities	20,274 (26.9%)	5,160 (22.4%)	15,114 (28.9%)	<0.001
No. of influenza vaccines in 2014–18				<0.001
0	17,984 (23.9%)	11,503 (50.0%)	6,481 (12.4%)	
1–3	25,382 (33.7%)	7,506 (32.7%)	17,876 (34.2%)	
4 or more	31,898 (42.4%)	3,978 (17.3%)	27,920 (53.4%)	
**Description of pneumococcal vaccination** No. of PPV23 doses received 2000–2018				
1			31,777 (60.8%)	
2+			20,500 (39.2%)	
Vaccinated at baseline			41,203 (78.8%)	
Vaccinated during follow-up (2014–2018)			11,074 (21.2%)	
The last dose received within 5 years of baseline or during follow-up			35,292 (67.5%)	

During follow-up, a total of 40,246 ARI events, 19,543 ARI-related antibiotic prescriptions, 12,777 LRTI events, and 6,268 LRTI-related antibiotic prescriptions were recorded in 75,264 individuals over an average of 3.4 person-years of follow-up. The majority of those who had an outcome of interest, only had one event reported during the four-year study period. The overall incidence of ARI, ARI-antibiotic, LRTI, and LRTI-antibiotic was 157.6, 76.0, 49.6, and 24.3 per 1000 person-years, respectively (data not shown). For every outcome, individuals who had ever been vaccinated showed a higher incidence than those who had never been vaccinated. Table 2 shows the follow-up time and incidence of study outcomes, stratified by vaccination status.

**Table 2:** Incidence of acute respiratory tract infections (ARIs), ARI-related antibiotic prescription, lower respiratory tract infection (LRTIs), and LRTI-related antibiotic prescription during follow-up (2014–2018) among patients aged 65 years or above, Australia.

	Acute respiratory tract infections (ARIs)		ARI-related antibiotic prescription		Lower respiratory tract infection (LRTIs)		LRTI-related antibiotic prescription	
	Never vaccinated	Ever vaccinated	Never vaccinated	Ever vaccinated	Never vaccinated	Ever vaccinated	Never vaccinated	Ever vaccinated
Total follow-up time (person-years)	84396	170972	84904	172097	85074	172485	85220	172845
Total no. of events	12602	27644	6127	13416	3455	9322	1710	4558
Incidence per 1000 person-years (95% CI)	149.3 (146.7–151.9)	161.7 (159.8–163.6)	72.2 (70.4–74.0)	78.0 (76.6–79.3)	40.6 (39.3–42.0)	54.0 (53.0–55.2)	20.1 (19.1–21.0)	26.4 (25.6–27.1)
No. of patients with at least one event (% of total)	7781 (23.1)	15989 (30.6)	4439 (13.2)	9354 (17.9)	2744 (8.1)	6756 (12.9)	1471 (4.4)	3680 (7.1)
Among patients with at least one event								
Only one event, n (%)	5133 (15.2)	9835 (18.8)	3344 (9.9)	6808 (13.0)	2232 (6.6)	5096 (9.8)	1277 (3.8)	3055 (5.9)
Two events, n (%)	1527 (4.5)	3449 (6.6)	744 (2.2)	1685 (3.2)	381 (1.1)	1135 (2.2)	161 (0.5)	456 (0.9)
Three or more events, n (%)	1121 (3.3)	2705 (5.2)	351 (1.0)	861 (1.7)	131 (0.4)	525 (1.0)	33 (0.1)	169 (0.3)

The associations of PPV23 vaccination with each study outcome are shown in Table 3. In the fully adjusted models, we did not find statistically significant associations of PPV23 vaccination with ARI events and ARI-related antibiotic prescriptions. However, for LRTI events and LRTI-related antibiotic prescriptions, we observed a significant positive association with PPV23 vaccination. S6 Table presents the sensitivity analysis with a follow-up period limited to one year. This analysis shows HRs with similar directions and magnitudes for the incidence of ARIs and ARI-related antibiotic prescriptions as the main analysis. However, the association with reduced ARI incidence was borderline statistically significant in the sensitivity analysis, and associations of PPV23 vaccination with LRTI and LRTI-related antibiotic prescriptions were no longer statistically significant. We did find some evidence of a reduction in ARI presentations when time since vaccination was considered (Table 4). Compared to those without pneumococcal vaccination, those who received PPV23 within <5 years of baseline had a slightly reduced incidence of ARI (adjusted HR 0.96; 95% CI 0.94–0.98; p = 0.002), but this was not the case among those who received PPV23 ≥5 years prior to baseline where the risk was marginally increased (adjusted HR 1.04; 95% CI 1.01–1.07; p = 0.013). For other study outcomes, we found no evidence of a reduction in incidence by recency of PPV23 receipt but rather an increased risk associated with PPV23 vaccination more than five years prior to baseline (Table 4).

**Table 3:** Associations of PPV23 vaccination and incidence of acute respiratory tract infections (ARIs), ARI-related antibiotic prescription, lower respiratory tract infection (LRTIs), and LRTI-related antibiotic prescription among patients aged 65 years or above, Australia 2014–2018.

Outcomes	Age & sex-adjusted model		Fully adjusted model*	
	HR (95% CI)	P value	HR (95% CI)	P value
**Ever received PPV23**				
ARIs	1.06 (1.04–1.08)	<0.001	0.98 (0.95–1.00)	0.066
ARI-related antibiotic	1.08 (1.05–1.11)	<0.001	1.01 (0.97–1.04)	0.743
LRTIs	1.17 (1.12–1.22)	<0.001	1.08 (1.03–1.13)	<0.001
LRTI-related antibiotic	1.19 (1.12–1.26)	<0.001	1.09 (1.03–1.16)	0.004

*Hazard ratios (HRs) with 95% confidence intervals (Cis) were estimated by adjusting for age group, sex, remoteness of practice, socio-economic status, number of GP visits in 2012 & 2013, smoking status, flu vaccination status during the follow-up period, asthma, Chronic obstructive pulmonary disease (COPD), heart disease, chronic kidney disease, chronic liver disease, diabetes, and haematological malignancy

**Table 4:** Associations of time since PPV23 vaccination and incidence of acute respiratory tract infections (ARIs), ARI-related antibiotic prescription, lower respiratory tract infection (LRTIs), and LRTI-related antibiotic prescription among patients aged 65 years or above, Australia 2014–2018.

Time since receipt of PPV23	Fully adjusted* HR with 95% CI	P value
**ARIs**		
Unvaccinated	1.00 (Ref)	
Vaccinated <5 years	0.96 (0.94–0.98)	0.002
Vaccinated ≥5 years	1.04 (1.01–1.07)	0.013
**ARI-related antibiotic**		
Unvaccinated	1.00 (Ref)	
Vaccinated <5 years	0.99 (0.96–1.03)	0.648
Vaccinated ≥5 years	1.09 (1.04–1.14)	<0.001
**LRTI**		
Unvaccinated	1.00 (Ref)	
Vaccinated <5 years	1.04 (0.99–1.09)	0.142
Vaccinated ≥5 years	1.15 (1.09–1.21)	<0.001
**LRTI-related antibiotic**		
Unvaccinated	1.00 (Ref)	
Vaccinated <5 years	1.07 (1.00–1.14)	0.058
Vaccinated ≥5 years	1.20 (1.11–1.30)	<0.001

* Hazard ratios (HRs) with 95% confidence intervals (Cis) were estimated by adjusting for age group, sex, remoteness of practice, socio-economic status, number of GP visits in 2012 & 2013, smoking status, flu vaccination status during the follow-up period, asthma, COPD, heart disease, chronic kidney disease, chronic liver disease, diabetes, and haematological malignancy

In stratified analyses, we found that PPV23 was associated with a reduced likelihood of ARI events among those who did not have comorbidities (HR 0.96, 95% CI 0.94–0.99) but not among those with comorbidities (HR 1.02, 95% CI 0.98–1.07) (Table 5). We found no evidence of a difference in the association of PPV23 with the outcomes by smoking status. Sensitivity analyses with negative control outcomes showed that PPV23 vaccination was not associated with UTI (adjusted HR 1.01, 95% CI 0.96–1.06), but there was a small yet statistically significant positive association with gastroenteritis (adjusted HR 1.09, 95% CI 1.00–1.19) (S5 Table).

**Table 5:** Associations of PPV23 vaccination and incidence of acute respiratory tract infections (ARIs), ARI-related antibiotic prescription, lower respiratory tract infection (LRTIs), and LRTI-related antibiotic prescription among patients aged 65 years or above according to comorbidity status and smoking status, Australia 2014–2018.

Outcomes	Adjusted* HR (95% CI)			
	No comorbidity	Comorbidity**	Never smoker	Ever smoker
ARIs	0.96 (0.94–0.99)	1.02 (0.98–1.07)	1.00 (0.97–1.04)	0.97 (0.93–1.00)
ARI-related antibiotic	0.99 (0.95–1.03)	1.06 (0.99–1.13)	1.04 (0.99–1.09)	0.98 (0.93–1.04)
LRTIs	1.05 (0.99–1.11)	1.13 (1.06–1.22)	1.11 (1.05–1.18)	1.09 (1.02–1.16)
LRTI-related antibiotic	1.07 (0.99–1.16)	1.14 (1.03–1.26)	1.13 (1.04–1.23)	1.10 (1.01–1.21)

* Hazard ratios (HRs) with 95% confidence intervals (CIs) were estimated by adjusting for age group, sex, remoteness of practice, socio-economic status, number of GP visits in 2012 & 2013, smoking status, and flu vaccination status during the follow-up period, as appropriate.

**Comorbidity: patients were classified as having at least one comorbid condition if there was a corresponding record in the two years prior (2012 and 2013) to the follow-up period.

## Discussion

In this population-based cohort of Australian adults aged ≥65 years attending primary care, we observed a small reduction in the incidence of ARI associated with PPV23 vaccination, but this was only significant among those who were vaccinated within five years of study baseline (~4% reduction, p = 0.002). However, PPV23 vaccination was not found to reduce in the incidence of ARI-related antibiotic prescription, LRTI, and LRTI-related antibiotic prescription. Stratified analysis also suggested that this reduction in incidence of ARI with PPV23 vaccination only occurred among those without comorbidities.

We found that 70% of all patients aged ≥65 years had at least one dose of PPV23 between 2000 and the end of the study period (i.e., 2018). A recent study based on the MedicineInsight data showed that 69% of those aged 60–65 years in 2010 had a recorded pneumococcal vaccination by 2017, with a peak age of vaccination at 66 years [18]. Similar to our study, they also reported that pneumococcal vaccination was more likely among those with comorbidities, ex-smokers, and frequent GP attendees. It is important to understand that recommendations for pneumococcal vaccination among adults have changed significantly over time in Australia, which in turn can impact vaccination coverage [13]. For example, a systematic review of Australian studies from 1992–2013 reported that pneumococcal vaccination coverage in people ≥65 years increased from 35.4% prior to 2005 to 56.0% from 2005 onwards, after the introduction of the free vaccine in the National Immunisation Programme (15). Pneumococcal vaccination uptake in our study cohort was similar to that found among patients aged 65 years or over in England (70%) [24].

In our study, recent vaccination with PPV23, that is within 5 years of study baseline, was associated with a significant reduction in ARI incidence (HR 0.96, 95% CI 0.94–0.98) but that was not observed when vaccination was received over 5 years ago. While we could not find any earlier studies of PPV23 vaccine on ARI incidence, previous studies have shown that the effectiveness of PPV23 vaccine against other outcomes, for example, invasive pneumococcal disease, also declined with time since vaccination [3, 5, 6]. We also found that this association between PPV23 and ARI incidence was significant only among those without comorbidities (HR 0.96, 95% CI 0.94–0.99). A potential explanation for this finding might be that comorbid conditions in older adults can be associated with poorer immune response to vaccine [25]. The small reduction in ARI incidence that we found might be expected as PPV23 is specifically targeted for the prevention of infections caused by *Streptococcus pneumoniae* but not infections caused by other microorganisms. Even though the estimated reduction in ARI incidence in our study was small, the high incidence of ARI in primary care in older adults (157.6 cases per 1000 person-year) suggests vaccination could still lead to a substantial reduction in GP presentations at the population level.

We found PPV23 receipt was associated with LRTI presentation in primary care settings rather than a reduction in risk, although this was predominantly found among those who received their vaccine more than five years prior to baseline. Previous studies examining the effectiveness of PPV23 given in the last five years against LRTI conditions in hospital settings showed no significant reduction in the incidence of all-cause pneumonia [26] and community-acquired pneumonia [27], and a Belgian study based on GP registry data did not find any protective effect of either PPV23 or PCV13 against LRTI [8]. However, this study did find individuals who had received both vaccines had reduced risk. Our findings, when confined to recent receipt of PPV23, are consistent with these earlier studies. As higher-risk individuals were more likely to receive PPV23 vaccine, the association of PPV23 given more than five years earlier with LRTI compared to unvaccinated populations could reflect this propensity to vaccine higher-risk populations.

In our study population, we found that nearly half of all ARI and LRTI presentations were associated with antibiotic prescribing. Previous Australian studies reported that antibiotics are prescribed in 32% and 70% of GP consultations for upper respiratory tract infection (URTI) and “other” respiratory infection, respectively [28, 29]. Studies from other countries also reported high use of antibiotics for ARIs and LRTIs in GP settings [30–32]. However, we found no evidence of association between PPV23 vaccination and ARI-related antibiotic prescriptions, LRTI, and LRTI-related antibiotic prescriptions. An analysis based on data from the Community-Acquired Pneumonia immunization Trial in Adults (CAPiTA) also did not find any association between PCV13 and LRTI-related antibiotic use in primary care [9].

The strengths of our study include the use of electronic health records from a longitudinal general practice database, which includes detailed vaccination, ARI events, and antibiotic prescription records. We also had information on various clinical and patient characteristics to adjust for potential confounders in our analysis and included negative control outcomes. Our study has several limitations. First, we ascertained the diagnosis of ARI and LRTI outcomes from GP electronic records and did not have radiography or laboratory reports to confirm the diagnosis. Additionally, because we relied on primary care records, we could not capture all ARI and LRTI outcomes, particularly severe cases that required hospitalisation. This could lead to misclassification of the outcomes. Third, we defined comorbid conditions based on a pre-coded conditions flag in the MedicineInsight dataset which did not specify the timing of diagnosis. Therefore, we could not properly identify someone’s risk of pneumococcal disease prior to age 65, which may have increased their likelihood of being vaccinated prior to age 65. Fourth, some patients might be vaccinated or diagnosed in primary care services not included in the MedicineInsight data network. Finally, residual confounding may still exist due to insufficient adjustment or unmeasured variables, as evidenced by our analyses. Differences in outcomes between the main analysis, averaging 3.4 years of follow-up, and the sensitivity analysis, limited to 1 year, could be explained by residual confounding by age which was not time-updated in the main analyses. The older adults were more likely to have ever received PPV23 than younger adults and age will affect outcomes. Other explanations for the differences could be related to the smaller numbers in the sensitivity analyses that could explain the lack of significant effect for PPV23 and LRTI and LRTI-related antibiotic prescriptions.

In summary, in this large population-based study, we found that recent PPV23 vaccination may lead to a small reduction in the incidence of ARI presentations in older adults, but PPV23 vaccination was not associated with clinically relevant reductions in LRTIs and ARI and LRTI-related antibiotic prescriptions. These findings help us to understand the potential wider benefits of PPV23 vaccination and can inform relevant policy regarding pneumococcal vaccination programs in older adults.

## Supporting information

S1 Fig Schematic diagram of multiple events, vaccination status, and risk sets for four different subjects. (DOCX)

S1 File (DOCX)

S1 Table Comorbidities included from MedicineInsight conditions detail table. (DOCX)

S2 Table Terms used to identify pneumococcal vaccination (PPV23). (DOCX)

S3 Table Terms used to identify acute respiratory tract infection (ARI) and lower respiratory tract infection (LRTI). (DOCX)

S4 Table Terms used for identifying acute respiratory tract infection (ARI) and lower respiratory tract infection (LRTI) related antibiotic prescription. (DOCX)

S5 Table Hazard ratios comparing PPV23 vaccination to no vaccination for the outcomes of presentation for urinary tract infection (UTI) or gastroenteritis (negative control outcomes). (DOCX)

S6 Table Hazard ratios comparing PPV23 vaccination to no vaccination for study outcomes (follow up period limited to 1 year: 1 Jan 2014–31 Dec 2014). (DOCX)

## References

[1] Henriques-Normark B, Tuomanen EI. The pneumococcus: epidemiology, microbiology, and pathogenesis. Cold Spring Harbor perspectives in medicine. 2013;3(7):a010215. doi: 10.1101/cshperspect.a010215 23818515 PMC3685878

[2] Australian Institute of Health and Welfare. Pneumococcal disease in Australia. 2018.

[3] Berild JD, Winje BA, Vestrheim DF, Slotved H-C, Valentiner-Branth P, Roth A, et al. A systematic review of studies published between 2016 and 2019 on the effectiveness and efficacy of pneumococcal vaccination on pneumonia and invasive pneumococcal disease in an elderly population. Pathogens. 2020;9(4):259. doi: 10.3390/pathogens9040259 32260132 PMC7238108

[4] Blommaert A, Hanquet G, Willem L, Theeten H, Thiry N, Bilcke J, et al. Use of pneumococcal vaccines in the elderly: an economic evaluation. 2016. 10.1080/21645515.2018.1428507 PMC5989887 29420161

[5] Falkenhorst G, Remschmidt C, Harder T, Hummers-Pradier E, Wichmann O, Bogdan C. Effectiveness of the 23-valent pneumococcal polysaccharide vaccine (PPV23) against pneumococcal disease in the elderly: systematic review and meta-analysis. PLoS One. 2017;12(1):e0169368. doi: 10.1371/journal.pone.0169368 28061505 PMC5218810

[6] Kraicer-Melamed H, O’Donnell S, Quach C. The effectiveness of pneumococcal polysaccharide vaccine 23 (PPV23) in the general population of 50 years of age and older: a systematic review and meta-analysis. Vaccine. 2016;34(13):1540–50. doi: 10.1016/j.vaccine.2016.02.024 26899372

[7] Lewnard JA, Bruxvoort KJ, Hong VX, Grant LR, Jódar L, Cané A, et al. Effectiveness of pneumococcal conjugate vaccination against virus-associated lower respiratory tract infection among adults: a case-control study. The Journal of Infectious Diseases. 2022. 10.1093/infdis/jiac098 PMC9383607 35323906

[8] Mamouris P, Henrard S, Molenberghs G, Verhaegen J, Lin G, Vaes B. Pneumococcal vaccination prevented severe LRTIs in adults: a causal inference framework applied in registry data. Journal of clinical epidemiology. 2022;143:118–27. doi: 10.1016/j.jclinepi.2021.12.008 34896235

[9] van Werkhoven CH, Bolkenbaas M, Huijts SM, Verheij TJ, Bonten MJ. Effects of 13-valent pneumococcal conjugate vaccination of adults on lower respiratory tract infections and antibiotic use in primary care: secondary analysis of a double-blind randomized placebo-controlled study. Clinical Microbiology and Infection. 2021;27(7):995–9. doi: 10.1016/j.cmi.2020.09.011 32971253

[10] Akhtar A, Hassali MAA, Zainal H, Ali I, Iqbal MS, Khan AH. Respiratory-tract infections among geriatrics: prevalence and factors associated with the treatment outcomes. Therapeutic Advances in Respiratory Disease. 2021;15:1753466620971141. doi: 10.1177/1753466620971141 33910420 PMC8108383

[11] O’Connor R, O’Doherty J, O’Regan A, O’Neill A, McMahon C, Dunne C. Medical management of acute upper respiratory infections in an urban primary care out-of-hours facility: Cross-sectional study of patient presentations and expectations. BMJ Open. 2019;9:e025396. doi: 10.1136/bmjopen-2018-025396 30772860 PMC6398638

[12] Renati S, Linder JA. Necessity of office visits for acute respiratory infections in primary care. Family practice. 2016;33(3):312–7. doi: 10.1093/fampra/cmw019 27048524 PMC4931816

[13] Australian Technical Advisory Group on Immunisation AGDoH. Public consultation on changes to the recommended use of pneumococcal vaccines. 2020.

[14] Brynley Hull AH, Aditi Dey, Julia Brotherton, Kristine Macartney, Frank Beard. NCIRS Annual Immunisation Coverage Report 2021. 2021. 10.33321/cdi.2023.47.47 37817316

[15] Dyda A, Karki S, Hayen A, MacIntyre CR, Menzies R, Banks E, et al. Influenza and pneumococcal vaccination in Australian adults: a systematic review of coverage and factors associated with uptake. BMC Infectious Diseases. 2016;16(1):1–15. doi: 10.1186/s12879-016-1820-8 27670446 PMC5037616

[16] Menzies R, Stein A, Booy R, Van Buynder P, Litt J, Cripps A. The impact of the changing pneumococcal national immunisation program among older Australians. Vaccine. 2021;39(4):720–8. doi: 10.1016/j.vaccine.2020.12.025 33384189

[17] Busingye D, Gianacas C, Pollack A, Chidwick K, Merrifield A, Norman S, et al. Data Resource Profile: MedicineInsight, an Australian national primary health care database. International journal of epidemiology. 2019;48(6):1741–h. doi: 10.1093/ije/dyz147 31292616

[18] Frank O, De Oliveira Bernardo C, González-Chica DA, Macartney K, Menzies R, Stocks N. Pneumococcal vaccination uptake among patients aged 65 years or over in Australian general practice. Human vaccines & immunotherapeutics. 2020;16(4):965–71. doi: 10.1080/21645515.2019.1682844 31634028 PMC7227629

[19] Peng Z, Hayen A, Liu B. Practice-and individual-level antibiotic prescribing associated with antibiotic treatment non-response in respiratory tract infections: a national retrospective observational study. Journal of Antimicrobial Chemotherapy. 2021;76(3):804–12. doi: 10.1093/jac/dkaa509 33575785

[20] Statistics ABO. Socio-economic indexes for areas (SEIFA). Canberra: Australian Bureau of Statistics. 2011.

[21] Pink B. Australian statistical geography standard (ASGS): volume 5—remoteness structure. Canberra: Australian Bureau of Statistics. 2011.

[22] He W-Q, Kirk M, Hall J, Liu B. 264 Antibiotic use associated with acute gastroenteritis in Australian primary care. International Journal of Epidemiology. 2021;50(Supplement_1):dyab168. 272.

[23] Peng Z, Hayen A, Hall J, Liu B. Microbiology testing and antibiotic treatment for urinary tract infections in general practice: a nationwide observational study. Infection. 2021;49(2):249–55. doi: 10.1007/s15010-020-01512-6 32862305

[24] Public Health England D. Pneumococcal polysaccharide vaccine (PPV) coverage report, England, April 2014 to March 2015. Health Prot Rep. 2019;13(39):1–11.

[25] Kwetkat A, Heppner HJ. Comorbidities in the elderly and their possible influence on vaccine response. Vaccines for Older Adults: Current Practices and Future Opportunities. 2020;43:73–85. doi: 10.1159/000504491 32305984

[26] Kolditz M, Schmitt J, Pletz MW, Tesch F. Impact of pneumococcal polysaccharide vaccine on incidence and mortality after pneumonia in adults aged≥ 60 years—a population-based retrospective cohort study. Clinical Microbiology and Infection. 2018;24(5):500–4. 28830805 10.1016/j.cmi.2017.08.010

[27] Domínguez À, Soldevila N, Toledo D, Torner N, Force L, Pérez MJ, et al. Effectiveness of 23-valent pneumococcal polysaccharide vaccination in preventing community-acquired pneumonia hospitalization and severe outcomes in the elderly in Spain. PLoS One. 2017;12(2):e0171943. doi: 10.1371/journal.pone.0171943 28187206 PMC5302444

[28] Fletcher-Lartey S, Khan R. Perceptions of barriers to the management of respiratory tract infections in general practice settings in Australia. Australian Journal of Primary Health. 2017;23(5):471–5. doi: 10.1071/PY17017 28931456

[29] Sargent L, McCullough A, Del Mar C, Lowe J. Is Australia ready to implement delayed prescribing in primary care?: a review of the evidence. Australian family physician. 2016;45(9):688–90. 27606375

[30] Baillie EJ, Merlo G, Magin P, Tapley A, Mulquiney KJ, Davis JS, et al. Antibiotic prescribing for upper respiratory tract infections and acute bronchitis: a longitudinal analysis of general practitioner trainees. Family Practice. 2022. doi: 10.1093/fampra/cmac052 35640041 PMC9680663

[31] Little P, Francis NA, Stuart B, O’Reilly G, Thompson N, Becque T, et al. Antibiotics for lower respiratory tract infection in children presenting in primary care in England (ARTIC PC): a double-blind, randomised, placebo-controlled trial. The Lancet. 2021;398(10309):1417–26. 10.1016/S0140-6736(21)01431-8 PMC8542731 34562391

[32] Zhuo C, Wei X, Zhang Z, Hicks JP, Zheng J, Chen Z, et al. An antibiotic stewardship programme to reduce inappropriate antibiotic prescribing for acute respiratory infections in rural Chinese primary care facilities: study protocol for a clustered randomised controlled trial. Trials. 2020;21(1):1–14. 32398065 10.1186/s13063-020-04303-4 PMC7216131

