# Supplementary material for: Pneumococcal vaccination and primary care presentations for acute respiratory tract infection and antibiotic prescribing in older adults
Source: PLoS One. 2024 Apr 18;19(4):e0299924. doi: 10.1371/journal.pone.0299924 (PMC11025920; doi:10.1371/journal.pone.0299924)
Supplement: S1 Fig — (DOCX) [file pone.0299924.s001.docx]

**Supplementary methods**

**Organisation of data for analysis of time-varying vaccination status and multiple outcome events**

Figure S1 displays an illustrative scheme of four subjects' recurrent events, vaccination status, and risk sets. Subject 1 had a vaccination record during follow-up and had one event. Subject 2 had two events and, in between event one and two, was vaccinated. Subject 3 was vaccinated before baseline and had four events in total. Two essential features of recurrent event data are that the events are ordered, and the subject can only be at risk for one such event at a time.


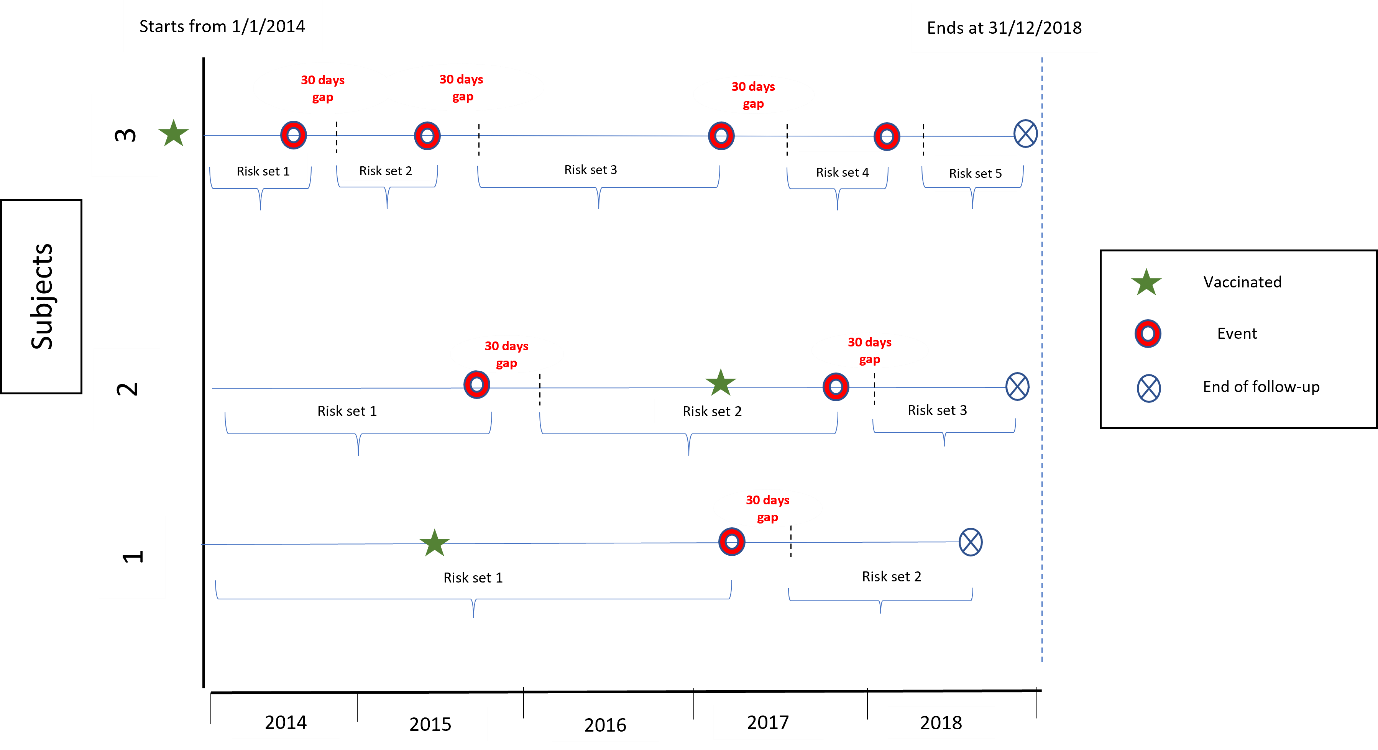


**Supplementary Figure S1.** **Schematic diagram of multiple events, vaccination status, and risk sets for four different subjects**

**Prentice, Williams, and Peterson Total Time (PWPTT) multiple failures survival model:** We used this model to estimate hazard ratios (HR) with 95% CI for all four outcomes (ARI, ARI-related antibiotic prescriptions, LRTI, and LRTI-related antibiotic drugs) comparing vaccinated vs. unvaccinated patients. This model evaluates the effect of a covariate for the kth event since the entry time in the study. In contrast to a standard Cox proportional hazard model, this model analyses ordered multiple events by stratification based on the previous number of events during the follow-up period. All participants are at risk for the first stratum, but only those with an event in the previous stratum are at risk for the successive one. This model allows the risk of having the outcome to vary for each subsequent event, for example, having had one ARI event it is likely that there is an increased risk of having a subsequent ARI event. (1).

Reference:

1. Amorim LD, Cai J. Modelling recurrent events: a tutorial for analysis in epidemiology. International journal of epidemiology. 2015;44(1):324-33.
