# Supplementary material for: Pneumococcal vaccination and primary care presentations for acute respiratory tract infection and antibiotic prescribing in older adults
Source: PLoS One. 2024 Apr 18;19(4):e0299924. doi: 10.1371/journal.pone.0299924 (PMC11025920; doi:10.1371/journal.pone.0299924)
Supplement: S1 File — (DOCX) [file pone.0299924.s002.docx]

**Pneumococcal vaccination and primary care presentations for acute respiratory tract infection and antibiotic prescribing in older adults**

Fariha Binte Hossain^1^, Sanjay Jayasinghe^2^, Katrina Blazek^1^, Wen-Qiang He^3^, Bette Liu^1,2^

**Supplementary materials**

**Supplementary methods**

**Organisation of data for analysis of time-varying vaccination status and multiple outcome events**

Figure S1 displays an illustrative scheme of four subjects' recurrent events, vaccination status, and risk sets. Subject 1 had a vaccination record during follow-up and had one event. Subject 2 had two events and, in between event one and two, was vaccinated. Subject 3 was vaccinated before baseline and had four events in total. Two essential features of recurrent event data are that the events are ordered, and the subject can only be at risk for one such event at a time.


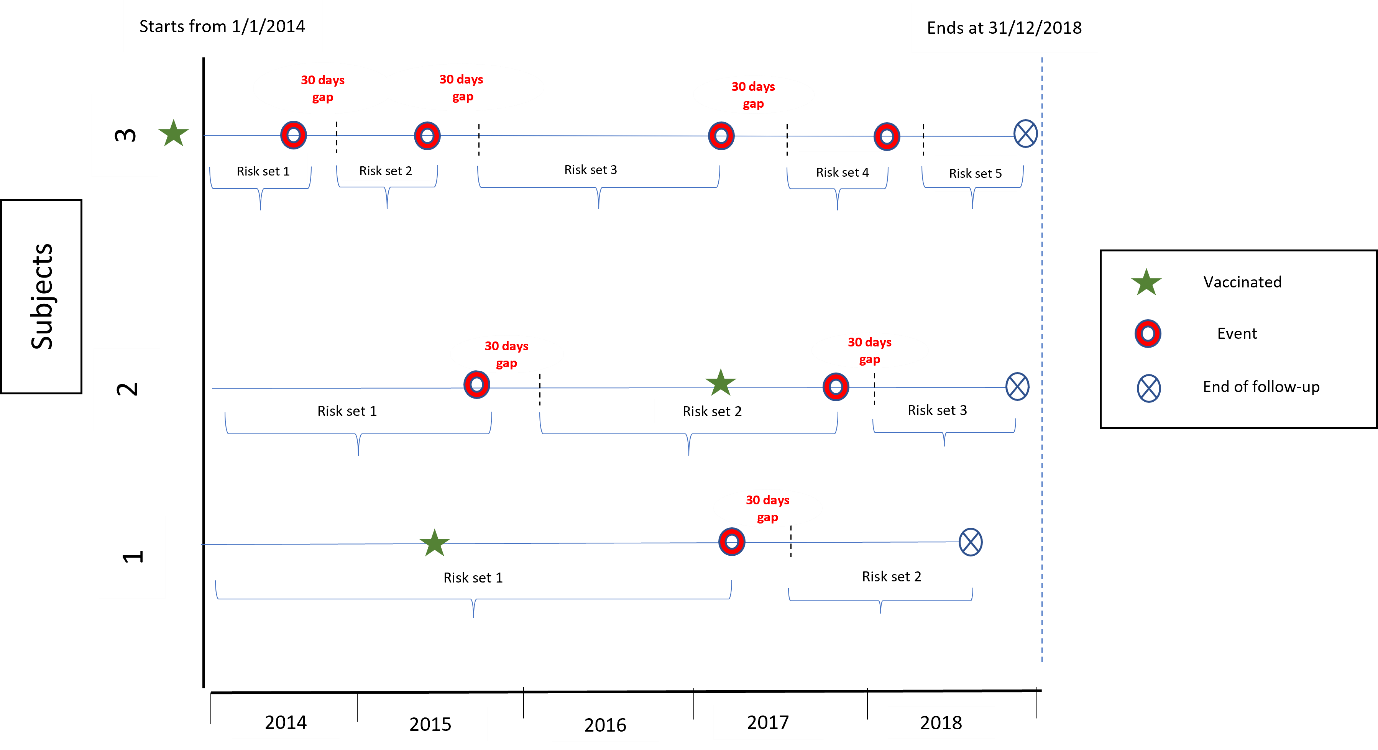


**Supplementary Figure S1.** **Schematic diagram of multiple events, vaccination status, and risk sets for four different subjects**

**Prentice, Williams, and Peterson Total Time (PWPTT) multiple failures survival model:** We used this model to estimate hazard ratios (HR) with 95% CI for all four outcomes (ARI, ARI-related antibiotic prescriptions, LRTI, and LRTI-related antibiotic drugs) comparing vaccinated vs. unvaccinated patients. This model evaluates the effect of a covariate for the kth event since the entry time in the study. In contrast to a standard Cox proportional hazard model, this model analyses ordered multiple events by stratification based on the previous number of events during the follow-up period. All participants are at risk for the first stratum, but only those with an event in the previous stratum are at risk for the successive one. This model allows the risk of having the outcome to vary for each subsequent event, for example, having had one ARI event it is likely that there is an increased risk of having a subsequent ARI event. (1).

**Methods for identifying chronic conditions**

MedicineInsight database custodians designed coding algorithms to identify patients with specific health conditions. These algorithms use data from any of three electronic health record (EHR) fields available from the source practice information systems: diagnosis, reason for visit, and reason for prescription. Each field contains either user-selected terms with associated codes from a drop-down list within the EHR software or free text entries. The algorithms classify a patient as having a particular health condition if a coded term or a text string from a predefined list has been recorded at least once for that patient in any of the three fields (2).

**S1 Table.** **Comorbidities included from MedicineInsight conditions detail table**

| Comorbidities | Variable names and description |
| --- | --- |
| Chronic Heart Disease | f_CHD_ATH: Coronary Heart Disease and Atherosclerosis  f_CHD_ATH_PR: Coronary Heart Disease and Atherosclerosis Related Procedure  f_CHD_ATH_TEST: Coronary Heart Disease and Atherosclerosis Test  f_CHD_PR: Coronary Heart disease Related Procedure  f_CHD_RA: Coronary Heart Disease Related Activity  f_HF: Heart Failure |
| Chronic Lung Disease |  |
| 1. Asthma | f_ASTH: Asthma |
| 1. Chronic obstructive pulmonary disease | f_COPD: Chronic Obstructive Pulmonary Disease |
| Chronic liver disease | f_CLD: Chronic Liver Disease |
| Chronic kidney disease | f_CKD_1: Chronic Kidney Disease - Stage 1  f_CKD_2: Chronic Kidney Disease - Stage 2  f_CKD_3: Chronic Kidney Disease - Stage 3  f_CKD_4: Chronic Kidney Disease - Stage 4  f_CKD_5: Chronic Kidney Disease - Stage 5  f_CKD_UNSP: Chronic Kidney Disease – Unspecified  f_CRF: Chronic Renal Failure |
| Type 2 diabetes | f_DM_T2: Diabetes Mellitus Type 2 |
| Haematological malignancy (HM) | Free text search from variable “term” in the dataset |
|  | Acute Myeloid leukaemia |
|  | Acute Lymphoblastic leukaemia |
|  | Chronic Myeloid leukaemia |
|  | Chronic Lymphoblastic leukaemia |
|  | Multiple Myeloma |

*****Conditions flags are generated using algorithms that examine both coded and free-text data from the ‘Diagnosis’, ‘Reason for encounter’, and ‘Reason for prescription’ fields.

**S2 Table. Terms used to identify pneumococcal vaccination (PPV23).**

| Fields used for searching | Terms for inclusion | Terms for exclusion |
| --- | --- | --- |
| “Vaccine name” and “vaccine date” fields of the immunization dataset |  |  |
|  |  |  |
| Search strategy |  |  |
| We used vaccine brand names as well as a combination of all plausible terms to capture GP encounters related to pneumococcal vaccination | “Pn eumovax”, “pnemovax”, “pnemovax 23”, “pneomovax”, “pneukmovax”, “pneumovax”, “pneumovax 2005”, “pneumovax 1”, “pneumovax-booster”, “pneumovax 2002 and 2007” , “pneumovax 2002 and 2008”, “pneumovax given”, “pneumovax in 2009”, “pneumovax 23 0.5 MLS”, “penumovax 23 # 2 ”, “pneumovax 23 (2^nd^ dose)”, “pneumovax 23 1st”, “pneumovax 23 dose 2”, “pneumovax private”, “pneumovax 23L0018”, “pneumovax 23 dose 1 given by previous”, “pneumovax x 2 in lifetime so complete”, “pneumovax1”, “pneumovax no 2”, “pneumovax 23-2nd”, “pneumococcus (23 Valent)”, “pneumonovax”, “pneumonvax”. “pneumoovax”, “pneumov”, “pnumovax”, “pneumvax”, “pneumvax 23”, “pneuovax”, “pneunovax”, | “?”,“no”, “re”, “not”, “book”, “gone”, “seek”, “need”, “been”, “will”, “want”, “never”, “avoid”, ”react”, “await”, “reject”, “refuse”, “review”, “recall”, “record”, “remind”, “screen”, “request”, “appoint”, “history”, “already”, “attempt”, “discuss”, “decline”, “previous”, “immunity”, “recommend”, “side effect” |

**S3 Table. Terms used to identify acute respiratory tract infection (ARI) and lower respiratory tract infection (LRTI)**

| Fields used for searching | Terms for inclusion | Terms for exclusion |
| --- | --- | --- |
| “Encounter reason”, “Diagnosis reason”, and  “Prescription reason” fields in the corresponding datasets |  |  |
|  |  |  |
| Search strategy | For ARI |  |
| A combination of relevant medical terminologies and shorthand abbreviations was used to capture encounters related to ARI and LRTI | “sinusitis”, otitis media”, “tonsilitis”, “pharyngitis”, “laryngitis”, “pneumonia”, “upper respiratory tract infection”, “URTI” | allergic, ?, rhinosinusitis, rhinsinusitis, dressing change, fungal, viral, aspergillus, vit b12 injection, ecchymoses, herpatic, radiation, recovered, thrush, laryngoscopy, urticaria, pigmentosa, pressure, examinations all normal, LRTI gone, follow up, in case, improving, resolving, review, delete, recovery, improved, referral, viral, aspiration, mycoplasma, aspergillus, moraxella, legionella, chlamydial, chlamydia, prevention, cryptogenic, vaccination, resolved, atypical, ?, examinations all normal, gone, haemophilus, influenzae, review, residual, residula, staph aureus, e.coli, lichen sclerosis, interstitial, rib fractures, pneumocysstic, bronchiolitis, organizing, confusion, fall, eosinophilic, flu, vaccine, hyperferritinaemia, fatty liver, seizures, delirium, improving, improve, influenza, likely, nstemi, shot, necrotising, recovered, suspected, stenophomonas kleb. |
|  | For LRTI |  |
|  | “pneumonia”, “lower respiratory tract infection”, “LRTI”, “lrti” | allergic, ?, rhinosinusitis, rhinsinusitis, dressing change, fungal, viral, aspergillus, vit b12 injection, ecchymoses, herpatic, radiation, recovered, thrush, laryngoscopy, urticaria, pigmentosa, pressure, examinations all normal, LRTI gone, follow up, in case, improving, resolving, review, delete, recovery, improved, referral, viral, aspiration, mycoplasma, aspergillus, moraxella, legionella, chlamydial, chlamydia, prevention, cryptogenic, vaccination, resolved, atypical, ?, examinations all normal, gone, haemophilus, influenzae, review, residual, residula, staph aureus, e.coli, lichen sclerosis, interstitial, rib fractures, pneumocysstic, bronchiolitis, organizing, confusion, fall, eosinophilic, flu, vaccine, hyperferritinaemia, fatty liver, seizures, delirium, improving, improve, influenza, likely, nstemi, shot, necrotising, recovered, suspected, stenophomonas kleb. |

**S4 Table****. Terms used for identifying acute respiratory tract infection (ARI) and lower respiratory tract infection (LRTI) related antibiotic prescription**

| Fields used for searching | Terms for inclusion |
| --- | --- |
| “Medicine active ingredient” field of the prescription dataset | For both ARI and LRTI-related antibiotic prescription |
|  |  |
| Search strategy |  |
| We used combinations of search terms for systematic antibiotics commonly prescribed to treat respiratory illnesses | “Penicillin”, “doxycycline”, “nitrofurantoin”, amoxicillin, ampicillin, ciprofloxacin, azithromycin, flucloxacillin, trimethoprim, norfloxacin, clarithromycin, pheneticillin, levofloxacin, moxifloxacin, minocycline, erythromycin, ofloxacin, tetracycline, methenamine, cefuroxime, cefaclor, phenoxymethylpenicillin, cefalexin, roxithromycin, ceftriaxone, ceftibuten, linezolid, vancomycin, benzylpenicillin, metronidazole, chloramphenicol, dicloxacillin, clindamycin, lincomycin, cefalotin, cefazolin, ceftazidime, meropenem, ertapenem, fosfomycin, daptomycin, Colistin, tinidazole, Teicoplanin, lincomycin, tobramycin, tigecycline, piperacillin, ticarcillin, cloxacillin, tazobactam, cefoxitin, cefotaxime, cefepime, aztreonam, imipenem, sulfamethizole, sulfathiazole, sulfamethoxazole, sulfadiazine, spectinomycin, bacitracin, polymyxin, nalidixic, “gatifloxacin”, “gentamycin”, “neomycin”, “amikacin” |

**S5 Table. Hazard ratios comparing PPV23 vaccination to no vaccination for the outcomes of presentation for urinary tract infection (UTI) or gastroenteritis (negative control outcomes)**

| **Outcomes** | **Crude model** | | **Age- and sex-adjusted model** | | **Fully adjusted model*** | |
| --- | --- | --- | --- | --- | --- | --- |
|  | **HR (95%)** | **P value** | **HR (95%)** | **P value** | **HR (95%)** | **P value** |
| UTIs | 1.09 (1.05-1.13) | <0.001 | 1.02 (0.98-1.06) | 0.364 | 1.01 (0.97-1.06) | 0.640 |
| Gastroenteritis | 1.21 (1.12-1.31) | <0.001 | 1.24 (1.14-1.34) | <0.001 | 1.09 (1.00-1.19) | 0.042 |

*Adjusted for age group, sex, remoteness of practice, socio-economic status, number of GP visits in 2012 & 2013, smoking status, flu vaccination status during the follow-up period, asthma, COPD, heart disease, chronic kidney disease, chronic liver disease, diabetes, and haematological malignancy

**S6 Table. Hazard ratios comparing PPV23 vaccination to no vaccination for study outcomes (follow up period limited to 1 year: 1 Jan 2014 – 31 Dec 2014)**

| **Outcomes** | **Age & sex-adjusted model** | | **Fully adjusted model*** | |
| --- | --- | --- | --- | --- |
|  | HR (95% CI) | P value | HR (95% CI) | P value |
| **Ever received PPV23** |  |  |  |  |
| ARIs | 1.07 (1.02-1.11) | 0.002 | 0.96 (0.92-1.00) | 0.049 |
| ARI-related antibiotic | 1.08 (1.02-1.14) | 0.013 | 1.01 (0.95-1.08) | 0.715 |
| LRTIs | 1.17 (1.08-1.26) | 0.000 | 1.04 (0.96-1.13) | 0.338 |
| LRTI-related antibiotic | 1.15 (1.03-1.29) | 0.012 | 1.06 (0.94-1.19) | 0.381 |

*Adjusted for age group, sex, remoteness of practice, socio-economic status, number of GP visits in 2012 & 2013, smoking status, flu vaccination status during the follow-up period, asthma, COPD, heart disease, chronic kidney disease, chronic liver disease, diabetes, and haematological malignancy

References:

1. Amorim LD, Cai J. Modelling recurrent events: a tutorial for analysis in epidemiology. International journal of epidemiology. 2015;44(1):324-33.

2. Havard A, Manski-Nankervis J-A, Thistlethwaite J, Daniels B, Myton R, Tu K, et al. Validity of algorithms for identifying five chronic conditions in MedicineInsight, an Australian national general practice database. BMC Health Services Research. 2021;21(1):551.
