# Supplementary material for: Pneumococcal vaccination and primary care presentations for acute respiratory tract infection and antibiotic prescribing in older adults
Source: PLoS One. 2024 Apr 18;19(4):e0299924. doi: 10.1371/journal.pone.0299924 (PMC11025920; doi:10.1371/journal.pone.0299924)
Supplement: S1 Table — (DOCX) [file pone.0299924.s003.docx]

**Methods for identifying chronic conditions**

MedicineInsight database custodians designed coding algorithms to identify patients with specific health conditions. These algorithms use data from any of three electronic health record (EHR) fields available from the source practice information systems: diagnosis, reason for visit, and reason for prescription. Each field contains either user-selected terms with associated codes from a drop-down list within the EHR software or free text entries. The algorithms classify a patient as having a particular health condition if a coded term or a text string from a predefined list has been recorded at least once for that patient in any of the three fields (1).

**S1 Table.** **Comorbidities included from MedicineInsight conditions detail table**

| Comorbidities | Variable names and description |
| --- | --- |
| Chronic Heart Disease | f_CHD_ATH: Coronary Heart Disease and Atherosclerosis  f_CHD_ATH_PR: Coronary Heart Disease and Atherosclerosis Related Procedure  f_CHD_ATH_TEST: Coronary Heart Disease and Atherosclerosis Test  f_CHD_PR: Coronary Heart disease Related Procedure  f_CHD_RA: Coronary Heart Disease Related Activity  f_HF: Heart Failure |
| Chronic Lung Disease |  |
| 1. Asthma | f_ASTH: Asthma |
| 1. Chronic obstructive pulmonary disease | f_COPD: Chronic Obstructive Pulmonary Disease |
| Chronic liver disease | f_CLD: Chronic Liver Disease |
| Chronic kidney disease | f_CKD_1: Chronic Kidney Disease - Stage 1  f_CKD_2: Chronic Kidney Disease - Stage 2  f_CKD_3: Chronic Kidney Disease - Stage 3  f_CKD_4: Chronic Kidney Disease - Stage 4  f_CKD_5: Chronic Kidney Disease - Stage 5  f_CKD_UNSP: Chronic Kidney Disease – Unspecified  f_CRF: Chronic Renal Failure |
| Type 2 diabetes | f_DM_T2: Diabetes Mellitus Type 2 |
| Haematological malignancy (HM) | Free text search from variable “term” in the dataset |
|  | Acute Myeloid leukaemia |
|  | Acute Lymphoblastic leukaemia |
|  | Chronic Myeloid leukaemia |
|  | Chronic Lymphoblastic leukaemia |
|  | Multiple Myeloma |

*****Conditions flags are generated using algorithms that examine both coded and free-text data from the ‘Diagnosis’, ‘Reason for encounter’, and ‘Reason for prescription’ fields

Reference:

1. Havard A, Manski-Nankervis J-A, Thistlethwaite J, Daniels B, Myton R, Tu K, et al. Validity of algorithms for identifying five chronic conditions in MedicineInsight, an Australian national general practice database. BMC Health Services Research. 2021;21(1):551.
