# Supplementary material for: Pneumococcal vaccination and primary care presentations for acute respiratory tract infection and antibiotic prescribing in older adults
Source: PLoS One. 2024 Apr 18;19(4):e0299924. doi: 10.1371/journal.pone.0299924 (PMC11025920; doi:10.1371/journal.pone.0299924)
Supplement: S2 Table — (DOCX) [file pone.0299924.s004.docx]

**S2 Table. Terms used to identify pneumococcal vaccination (PPV23)**

| Fields used for searching | Terms for inclusion | Terms for exclusion |
| --- | --- | --- |
| “Vaccine name” and “vaccine date” fields of the immunization dataset |  |  |
|  |  |  |
| Search strategy |  |  |
| We used vaccine brand names as well as a combination of all plausible terms to capture GP encounters related to pneumococcal vaccination | “Pn eumovax”, “pnemovax”, “pnemovax 23”, “pneomovax”, “pneukmovax”, “pneumovax”, “pneumovax 2005”, “pneumovax 1”, “pneumovax-booster”, “pneumovax 2002 and 2007” , “pneumovax 2002 and 2008”, “pneumovax given”, “pneumovax in 2009”, “pneumovax 23 0.5 MLS”, “penumovax 23 # 2 ”, “pneumovax 23 (2^nd^ dose)”, “pneumovax 23 1st”, “pneumovax 23 dose 2”, “pneumovax private”, “pneumovax 23L0018”, “pneumovax 23 dose 1 given by previous”, “pneumovax x 2 in lifetime so complete”, “pneumovax1”, “pneumovax no 2”, “pneumovax 23-2nd”, “pneumococcus (23 Valent)”, “pneumonovax”, “pneumonvax”. “pneumoovax”, “pneumov”, “pnumovax”, “pneumvax”, “pneumvax 23”, “pneuovax”, “pneunovax”, | “?”,“no”, “re”, “not”, “book”, “gone”, “seek”, “need”, “been”, “will”, “want”, “never”, “avoid”, ”react”, “await”, “reject”, “refuse”, “review”, “recall”, “record”, “remind”, “screen”, “request”, “appoint”, “history”, “already”, “attempt”, “discuss”, “decline”, “previous”, “immunity”, “recommend”, “side effect” |
