# Supplementary material for: Pneumococcal vaccination and primary care presentations for acute respiratory tract infection and antibiotic prescribing in older adults
Source: PLoS One. 2024 Apr 18;19(4):e0299924. doi: 10.1371/journal.pone.0299924 (PMC11025920; doi:10.1371/journal.pone.0299924)
Supplement: S3 Table — (DOCX) [file pone.0299924.s005.docx]

**S3 Table. Terms used to identify acute respiratory tract infection (ARI) and lower respiratory tract infection (LRTI)**

| Fields used for searching | Terms for inclusion | Terms for exclusion |
| --- | --- | --- |
| “Encounter reason”, “Diagnosis reason”, and  “Prescription reason” fields in the corresponding datasets |  |  |
|  |  |  |
| Search strategy | For ARI |  |
| A combination of relevant medical terminologies and shorthand abbreviations was used to capture encounters related to ARI and LRTI | “sinusitis”, otitis media”, “tonsilitis”, “pharyngitis”, “laryngitis”, “pneumonia”, “upper respiratory tract infection”, “URTI” | allergic, ?, rhinosinusitis, rhinsinusitis, dressing change, fungal, viral, aspergillus, vit b12 injection, ecchymoses, herpatic, radiation, recovered, thrush, laryngoscopy, urticaria, pigmentosa, pressure, examinations all normal, LRTI gone, follow up, in case, improving, resolving, review, delete, recovery, improved, referral, viral, aspiration, mycoplasma, aspergillus, moraxella, legionella, chlamydial, chlamydia, prevention, cryptogenic, vaccination, resolved, atypical, ?, examinations all normal, gone, haemophilus, influenzae, review, residual, residula, staph aureus, e.coli, lichen sclerosis, interstitial, rib fractures, pneumocysstic, bronchiolitis, organizing, confusion, fall, eosinophilic, flu, vaccine, hyperferritinaemia, fatty liver, seizures, delirium, improving, improve, influenza, likely, nstemi, shot, necrotising, recovered, suspected, stenophomonas kleb. |
|  | For LRTI |  |
|  | “pneumonia”, “lower respiratory tract infection”, “LRTI”, “lrti” | allergic, ?, rhinosinusitis, rhinsinusitis, dressing change, fungal, viral, aspergillus, vit b12 injection, ecchymoses, herpatic, radiation, recovered, thrush, laryngoscopy, urticaria, pigmentosa, pressure, examinations all normal, LRTI gone, follow up, in case, improving, resolving, review, delete, recovery, improved, referral, viral, aspiration, mycoplasma, aspergillus, moraxella, legionella, chlamydial, chlamydia, prevention, cryptogenic, vaccination, resolved, atypical, ?, examinations all normal, gone, haemophilus, influenzae, review, residual, residula, staph aureus, e.coli, lichen sclerosis, interstitial, rib fractures, pneumocysstic, bronchiolitis, organizing, confusion, fall, eosinophilic, flu, vaccine, hyperferritinaemia, fatty liver, seizures, delirium, improving, improve, influenza, likely, nstemi, shot, necrotising, recovered, suspected, stenophomonas kleb. |
