# Supplementary material for: Pneumococcal vaccination and primary care presentations for acute respiratory tract infection and antibiotic prescribing in older adults
Source: PLoS One. 2024 Apr 18;19(4):e0299924. doi: 10.1371/journal.pone.0299924 (PMC11025920; doi:10.1371/journal.pone.0299924)
Supplement: S4 Table — (DOCX) [file pone.0299924.s006.docx]

**S4 Table****. Terms used for identifying acute respiratory tract infection (ARI) and lower respiratory tract infection (LRTI) related antibiotic prescription**

| Fields used for searching | Terms for inclusion |
| --- | --- |
| “Medicine active ingredient” field of the prescription dataset | For both ARI and LRTI-related antibiotic prescription |
|  |  |
| Search strategy |  |
| We used combinations of search terms for systematic antibiotics commonly prescribed to treat respiratory illnesses | “Penicillin”, “doxycycline”, “nitrofurantoin”, amoxicillin, ampicillin, ciprofloxacin, azithromycin, flucloxacillin, trimethoprim, norfloxacin, clarithromycin, pheneticillin, levofloxacin, moxifloxacin, minocycline, erythromycin, ofloxacin, tetracycline, methenamine, cefuroxime, cefaclor, phenoxymethylpenicillin, cefalexin, roxithromycin, ceftriaxone, ceftibuten, linezolid, vancomycin, benzylpenicillin, metronidazole, chloramphenicol, dicloxacillin, clindamycin, lincomycin, cefalotin, cefazolin, ceftazidime, meropenem, ertapenem, fosfomycin, daptomycin, Colistin, tinidazole, Teicoplanin, lincomycin, tobramycin, tigecycline, piperacillin, ticarcillin, cloxacillin, tazobactam, cefoxitin, cefotaxime, cefepime, aztreonam, imipenem, sulfamethizole, sulfathiazole, sulfamethoxazole, sulfadiazine, spectinomycin, bacitracin, polymyxin, nalidixic, “gatifloxacin”, “gentamycin”, “neomycin”, “amikacin” |
