# Supplementary material for: Pneumococcal vaccination and primary care presentations for acute respiratory tract infection and antibiotic prescribing in older adults
Source: PLoS One. 2024 Apr 18;19(4):e0299924. doi: 10.1371/journal.pone.0299924 (PMC11025920; doi:10.1371/journal.pone.0299924)
Supplement: S5 Table — (DOCX) [file pone.0299924.s007.docx]

**S5 Table. Hazard ratios comparing PPV23 vaccination to no vaccination for the outcomes of presentation for urinary tract infection (UTI) or gastroenteritis (negative control outcomes)**

| **Outcomes** | **Crude model** | | **Age- and sex-adjusted model** | | **Fully adjusted model*** | |
| --- | --- | --- | --- | --- | --- | --- |
|  | **HR (95%)** | **P value** | **HR (95%)** | **P value** | **HR (95%)** | **P value** |
| UTIs | 1.09 (1.05-1.13) | <0.001 | 1.02 (0.98-1.06) | 0.364 | 1.01 (0.97-1.06) | 0.640 |
| Gastroenteritis | 1.21 (1.12-1.31) | <0.001 | 1.24 (1.14-1.34) | <0.001 | 1.09 (1.00-1.19) | 0.042 |

*Adjusted for age group, sex, remoteness of practice, socio-economic status, number of GP visits in 2012 & 2013, smoking status, flu vaccination status during the follow-up period, asthma, COPD, heart disease, chronic kidney disease, chronic liver disease, diabetes, and haematological malignancy
