# Supplementary material for: Pneumococcal vaccination and primary care presentations for acute respiratory tract infection and antibiotic prescribing in older adults
Source: PLoS One. 2024 Apr 18;19(4):e0299924. doi: 10.1371/journal.pone.0299924 (PMC11025920; doi:10.1371/journal.pone.0299924)
Supplement: S6 Table — (DOCX) [file pone.0299924.s008.docx]

**S6 Table. Hazard ratios comparing PPV23 vaccination to no vaccination for study outcomes (follow up period limited to 1 year: 1 Jan 2014 – 31 Dec 2014)**

| **Outcomes** | **Age & sex-adjusted model** | | **Fully adjusted model*** | |
| --- | --- | --- | --- | --- |
|  | HR (95% CI) | P value | HR (95% CI) | P value |
| **Ever received PPV23** |  |  |  |  |
| ARIs | 1.07 (1.02-1.11) | 0.002 | 0.96 (0.92-1.00) | 0.049 |
| ARI-related antibiotic | 1.08 (1.02-1.14) | 0.013 | 1.01 (0.95-1.08) | 0.715 |
| LRTIs | 1.17 (1.08-1.26) | 0.000 | 1.04 (0.96-1.13) | 0.338 |
| LRTI-related antibiotic | 1.15 (1.03-1.29) | 0.012 | 1.06 (0.94-1.19) | 0.381 |

*Adjusted for age group, sex, remoteness of practice, socio-economic status, number of GP visits in 2012 & 2013, smoking status, flu vaccination status during the follow-up period, asthma, COPD, heart disease, chronic kidney disease, chronic liver disease, diabetes, and haematological malignancy
